# Supplementary figures and images for: Sublobar Resection Versus Lobectomy for Small (≤3 cm) NSCLC with Visceral Pleural Invasion: A Propensity-Score-Matched Survival Analysis from a Nationwide Cohort
Source: Cancers (Basel). 2025 Jun 14;17(12):1990. doi: 10.3390/cancers17121990 (PMC12191045; doi:10.3390/cancers17121990)

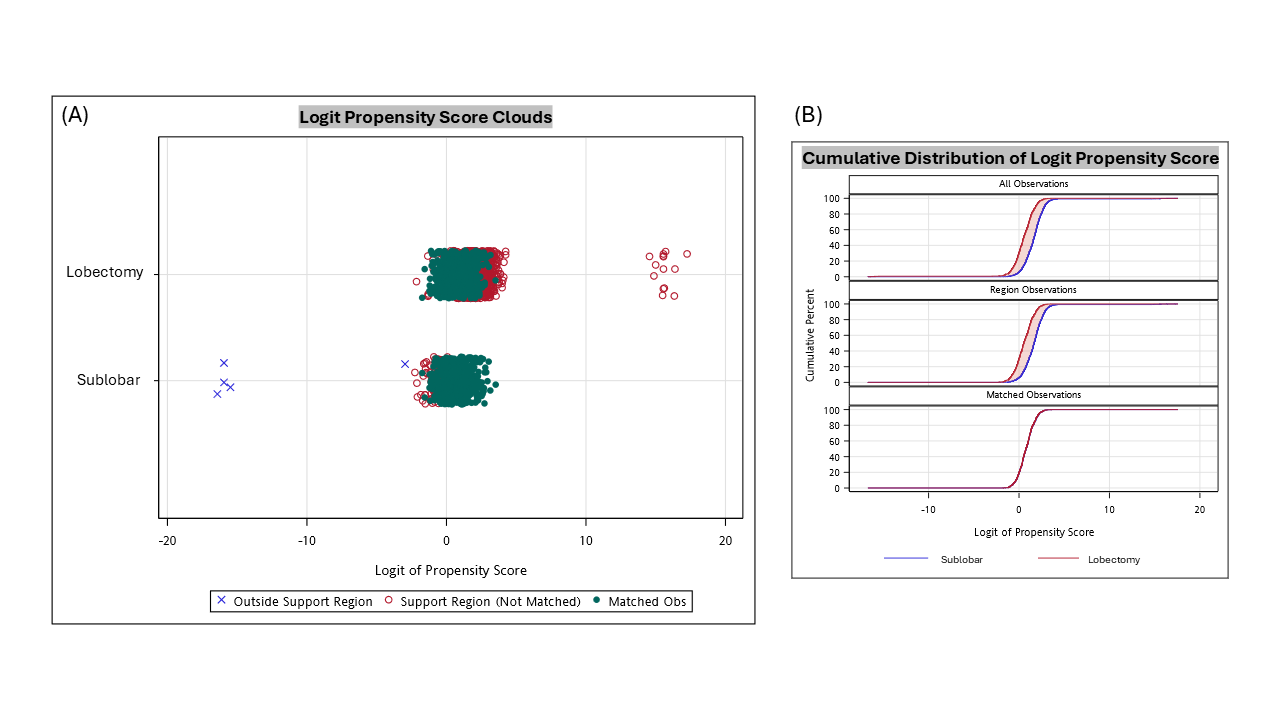

Supplement: Supplementary file 1 [file cancers-17-01990-s001.zip › Supplementary S1.png]

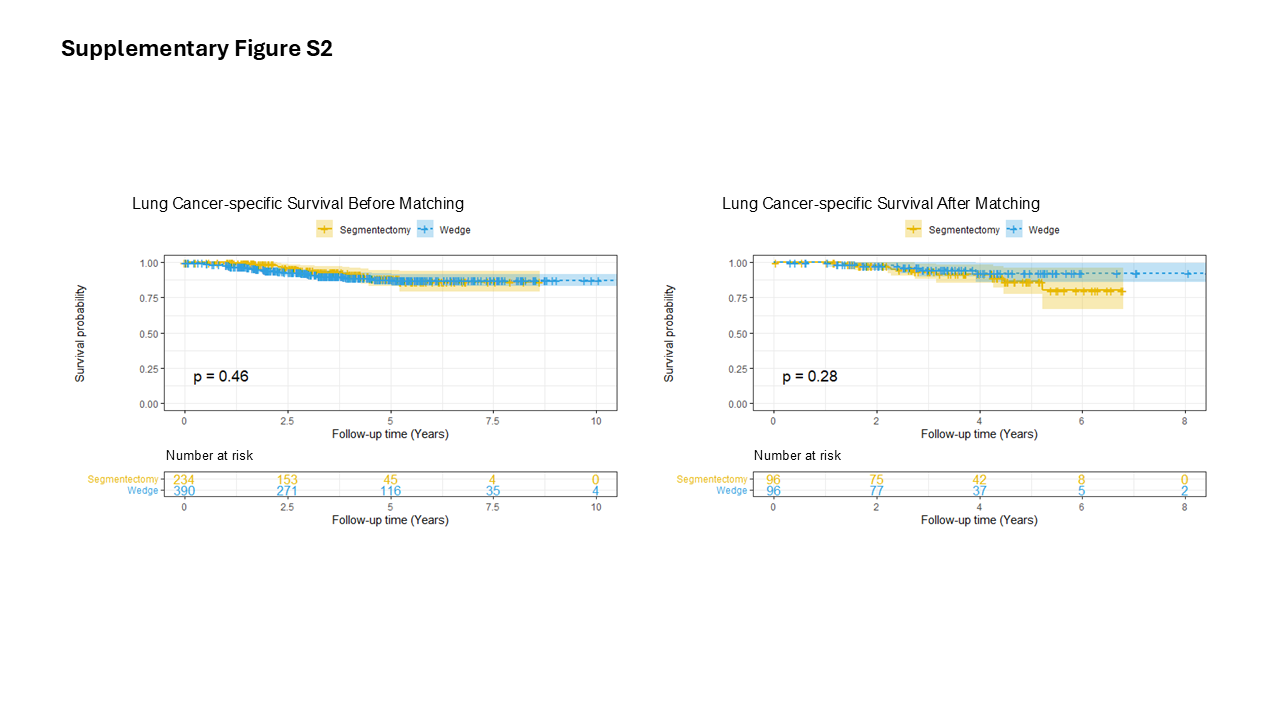

Supplement: Supplementary file 1 [file cancers-17-01990-s001.zip › Supplementary S2.png]
